# Maximizing research study effectiveness in malaria elimination settings: a mixed methods study to capture the experiences of field-based staff

**DOI:** 10.1186/s12936-017-2016-4

**Published:** 2017-09-11

**Authors:** Sara E. Canavati, Cesia E. Quintero, Britt Haller, Dysoley Lek, Sovann Yok, Jack S. Richards, Maxine Anne Whittaker

**Affiliations:** 1 Centre for Biomedical Research, Burnet Institute, Melbourne, Australia; 2 Vysnova Partners Inc., Washington, DC USA; 3 The Northern Hospital, Epping, Australia; 4 The National Centre for Parasitology, Entomology and Malaria Control, Ministry of Health, Phnom Penh, Cambodia; 5 Provincial Health Department, Pailin City, Pailin Province Cambodia; 6 Department of Medicine, The Peter Doherty Institute for Infection and Immunity, University of Melbourne, Melbourne, Australia; 7 Department of Infectious Diseases, Monash University, Melbourne, Australia; 8 College of Public Health, Medical and Veterinary Sciences, Division of Tropical Health and Medicine, James Cook University, Townsville, Australia

**Keywords:** Malaria, Mobile and migrant population, Clinical trials, Therapeutic efficacy studies, Artemisinin resistance, Study design, Multidrug resistant malaria, Malaria elimination, Cambodia, Malaria research, Operational research

## Abstract

**Background:**

In a drug-resistant, malaria elimination setting like Western Cambodia, field research is essential for the development of novel anti-malarial regimens and the public health solutions necessary to monitor the spread of resistance and eliminate infection. Such field studies often face a variety of similar implementation challenges, but these are rarely captured in a systematic way or used to optimize future study designs that might overcome similar challenges. Field-based research staff often have extensive experience and can provide valuable insight regarding these issues, but their perspectives and experiences are rarely documented and seldom integrated into future research protocols. This mixed-methods analysis sought to gain an understanding of the daily challenges encountered by research field staff in the artemisinin-resistant, malaria elimination setting of Western Cambodia. In doing so, this study seeks to understand how the experiences and opinions of field staff can be captured, and used to inform future study designs.

**Methods:**

Twenty-two reports from six field-based malaria studies conducted in Western Cambodia were reviewed using content analysis to identify challenges to conducting the research. Informal Interviews, Focus Group Discussions and In-depth Interviews were also conducted among field research staff. Thematic analysis of the data was undertaken using Nvivo 9^®^ software. Triangulation and critical case analysis was also used.

**Results:**

There was a lack of formalized avenues through which field workers could report challenges experienced when conducting the malaria studies. Field research staff faced significant logistical barriers to participant recruitment and data collection, including a lack of available transportation to cover long distances, and the fact that mobile and migrant populations (MMPs) are usually excluded from studies because of challenges in follow-up. Cultural barriers to communication also hindered participant recruitment and created unexpected delays. Field staff often paid a physical, emotional and financial cost, going beyond their duty in order to keep the study running.

**Conclusions:**

Formal monthly reports filled out by field study staff could be a key tool for capturing field study staff experiences effectively, but require specific report fields to encourage staff to outline their challenges and to propose potential solutions. Forging strong bonds with communities and their leaders may improve communication, and decrease barriers to participant recruitment. Study designs that make it feasible for MMPs to participate should be pursued; in addition to increasing the potential participant pool, this will ensure that the most malaria-endemic demographic is taken into account in research studies. Overlaps between clinical care and research create ethical dilemmas for study staff, a fact that warrants careful consideration. Lessons learned from study field staff should be used to create a set of locally-relevant recommendations to inform future study designs.

**Electronic supplementary material:**

The online version of this article (doi:10.1186/s12936-017-2016-4) contains supplementary material, which is available to authorized users.

## Background

The global eradication of malaria requires the continuous development of innovative diagnostic tools, treatment regimens and public health strategies [1–6]. All of this can only be achieved through research. The process of malaria elimination results in new barriers to conducting research that go beyond the often-experienced challenges inherent to research in remote locations. In the Greater Mekong Sub-region (GMS), where most countries are aiming to eliminate *Plasmodium falciparum* by 2020 [7], progress towards this goal has already brought about significant epidemiological shifts that present unique challenges, including a dramatic reduction in the incidence of malaria [8], and the clustering of cases in particular geographical areas and among specific groups [9]. Resistance of *P. falciparum* to both artemisinin and the partner drugs used in artemisinin-based combination therapy (ACT) in the region adds another layer of complexity [10–17].

Western Cambodia, a region located along the border with Thailand, is notable both for its sizable mobile and migrant population (MMP), who carry the bulk of the malaria burden in the GMS [18], and for historically being a frequent point of origin for drug resistance in *P. falciparum* [19–21]. Its highly mobile populations, compounded with particularly porous borders, facilitate the frequent importation and exportation of drug resistant parasites [22]. As is common in many malaria elimination settings, despite their higher burden of disease and their crucial role in its spread [23], MMPs in this region often slip through the cracks of passive malaria control measures aimed at the general population, and are often excluded from research studies because of the difficulty in conducting follow-up [24]. In a similar manner, participants who are labeled as “treatment failures” are also removed from the study and become ineligible for recruitment in future studies; in a drug-resistant setting like Western Cambodia, this is not infrequent. The combination of low malaria incidence [14], a mobile and remote participant pool [24], and high rates of participant ineligibility due to mobility and recrudescence, results in a number of barriers to effective field research.

In Western Cambodia, village malaria workers (VMWs) are vital members of malaria research teams, particularly when it comes to participant recruitment [25, 26]. Field-based research teams also include local physicians, lab technicians, nurses and other health workers. These health workers have extensive field-based experience and can provide valuable insight regarding the daily challenges in conducting that work [27]. Nevertheless, their perspectives are rarely accounted for in the design of research protocols, and their insights are largely absent from the scientific literature. This means that year after year, study designs do not take these perspectives into account and are therefore unable to overcome or attenuate recurrent context-specific barriers.

This paper presents the findings of a mixed methods assessment that was undertaken in order to understand the challenges faced by field workers conducting malaria research in Western Cambodia. There is no previous documentation of challenges faced by field staff in research elimination settings with low transmission and multi-drug resistance and malaria elimination. As the 2020 *P. falciparum* malaria elimination deadline draws nearer [28], there is a need to capture and learn from these experiences in order to improve the efficiency and quality of future studies. Given Western Cambodia’s unique position in the process of elimination and the development of *P. falciparum* resistance, this data can be useful in other GMS regions whose epidemiological context is evolving towards that of Western Cambodia.

## Methods

Research staff providing the data for this study included hospital laboratory staff, nurses, medical doctors, and Village Malaria Workers. A mixed methods approach was taken to data collection and analysis. The data collection period was between 2011 and 2014.

### Routine study reports and data collection

Data was collected from 22 routine study reports completed by field staff during five different field-based studies (Additional file 1: Table S1) carried out between 2012 and 2014 in rural areas of Western Cambodia. Four of the studies had used quarterly reports (17 quarterly reports). The fifth study employed monthly reports, and was in its sixth month (5 monthly reports). All reports had been filled out by the Study Coordinator or the Study Site Principal Investigator during periodic meetings in which all study staff were present, and were meant to record a consensus of all attendees. These studies were conducted in three provinces of Western Cambodia—Battambang, Pailin and Pursat. These were the only studies during the data collection period that were conducted in Western Cambodia, had written reports, and consent obtained to provide these written reports.

### Informal interviews of study field staff

Eleven informal interviews were conducted amongst study staff at the Pailin Referral Hospital. Participants consisted of doctors, laboratory workers, nurses and other staff working in on-going clinical trials at the time the study took place. The informal interviews were conducted during field visits or at the hospital. The interviews were structured as casual conversations centered around the topic of research staff experiences and the challenges they faced. The interviewer joined study activities and engaged in fieldwork to develop an understanding of the setting and to build rapport. Field notes were used to aid with recall. The validity and advantages of Informal Interview methodology has been well documented [29].

### In-depth interviews and focus group discussions and data collection

60 focus group discussions (FGDs) and 65 in-depth interviews (IDIs) were conducted among study staff, including VMWs and MMWs (mobile malaria workers) agreed to participate. For both FGDs and IDIs, the data collection instruments were based on previously-published methods [25], and adapted throughout the data collection period as necessary.

FGDs were conducted among VMWs and MMWs. In order to have sufficient participants for the farm-based FGDs, VMWs and MMWs were selected based on operational district rather than Health Centre (HC). Each VMW/MMW FGD consisted of eight participants; two male and two female FGDs were conducted per operational district.

IDIs were conducted among VMWs/MMWs and Health Centre staff. For both FDGs and IDIs, the HC staff, VMWs, MMWs were interviewed in each health centre.

#### Data analysis

FGDs and IDIs were recorded, fully transcribed, and translated into English. Data analysis consisted of examining, categorizing, tabulating or recombining the data. Thematic analysis around the key themes of the project was undertaken using Nvivo 9^®^ software and themes were identified and clustered to form overarching, larger themes. Triangulation and critical case analysis was also used.

#### Qualitative content analysis

Qualitative data gathered from the field reports were analyzed manually. The key categories of “challenges”, “delays”, “access”, “barriers”, “planning”, “transportation” and “recruitment” were used to manually code the reports [20]. The coded reports were then analysed using these key words as analytical categories to develop the following themes for discussion: participant recruitment and data collection, communication barriers, strain to field workers, and avenues to report challenges.

## Results

Overall, the study found that challenges faced by field research staff fell into the following categories: (i) logistical barriers to participant recruitment and data collection, (ii) cultural barriers to communication (iii) physical, emotional and financial strain to field workers, and (iv) a lack of avenues to report challenges.

### Demographics of study participants

Additional file 1: Table S2 shows the socio-demographic characteristics of the VMW assessment participants [25]. A total of 197 malaria volunteer workers, of which 163 were Village Malaria Workers and 34 were Mobile Malaria Workers, participated in this assessment. There was a greater percentage of male volunteers than female volunteers (58 and 42%). Their ages ranged from 17 to 68 years old, with a mean of 35 years. For most VMWs/MMWs, their primary profession was farming (92%). About half of them completed grade six, and more than a third of them had never attended school. Most of them (82%), reported being married or living with someone as married. The ethnicity for the majority of them was Khmer (98%). Over 80% of them had been working as a VMW/MMW for 2 years.

### Logistical barriers to participant recruitment and data collection

The data showed that logistical challenges were routine for field staff, and could be largely accounted for by a few main causes, such as the remote location of study sites, rainy season weather, and the epidemiological environment in the region.

Field staff reported that malaria patients could usually be found only in remote locations. Due to the need for random site selection, these locations were often far from each other.

The epidemiological context of the region was found to be a significant barrier to reaching required sample sizes. One field staff reported, *“it is difficult to recruit patients in the study because we need to recruit Plasmodium falciparum and Plasmodium mixed infections only, and the numbers are really low. Most patients are infected with Plasmodium vivax; hence we cannot include them in the study.”* IDI11

Another said, *“we didn’t reach the sample size even though we tried so hard. We found one participant, and he had mental problems and he wouldn’t follow instructions, so we had to exclude him. Another we could not include because he had HIV and he needed HIV treatment more urgently.”* IDI09

Although it was understood that the bulk of malaria infections would be found among MMPs, field staff could not access this potential participant pool: *“It is very difficult to follow up migrants. It is very difficult so we are told not to include them in studies.”* Recruiting sufficient participants within the scheduled time frame was therefore rarely feasible, and was reported to result in delays and cost over-runs. It also increased the distances that each staff member needed to cover in order to meet their quota.

Field staff would therefore be based permanently or temporarily in a remote location equipped with sample storage capacities, and then spend extensive periods of time travelling, sometimes under harsh weather, in order to conduct recruitment and follow-up activities. One field staff noted: “*I need to drive every day, very often, from village to village. Sometimes the whole day, or the whole night, even if it is raining. Sometimes I need to cross a whole mountain to get to the other village.”* IDI11

Another said, “*We have to go to their [the patients’] house. When we test their blood and confirm the disease, we give him them treatment. The following day and the day after that day we must go back to do the same things. We also have to ask if they feel better or what side effects they have. And their villages are far from us, so we spend so much time.”* FGD32

It was often reported that transportation was more time-consuming and costly than had originally been anticipated during the planning stages of projects, leading to budget shortages and missed deadlines. Many comments were made along the lines of the following: *“I had to go to remote area. The path became very muddy in the rainy season. My motorbike could not pass. We had to hire a truck to reach that area to provide drugs for participants. The truck got stuck climbing a big hill. We did not know whether by the sides of the pathway there were landmines or not. It was so difficult to reach that area, especially in the rainy season*.”

Landmines were reported to be a particular concern during the rainy season, as they can shift with the heavy rain and flooding; previously mine-free areas may no longer be safe. They also detonate more easily when the ground is soft. The remote locations and socioeconomic status of malaria patients created challenges for follow-up.

As one field staff explained, this was a significant factor in loss to follow-up: “*They might be away when we come to their village. Suppose we try to follow up 50 patients; we can only find 30 or 35. Because of this we miss the deadlines.*” IDI10

The transportation of biological samples was similarly complicated by long distances and the high temperatures, humidity and the unpredictability of the rainy season. A field staff member reported: *“Sometimes when we used to transport [samples] from Pursat field site to Pailin lab, the samples were already coagulated, because the car would get stuck in the mud. The ice would melt. By the time they arrived the samples were coagulated and we could not do anything.”* IDI15

Another commented: *“Sometimes the liquid nitrogen from Phnom Penh arrives late. When the nitrogen is late, the samples are sent late, and the samples can be damaged.”* IDI11

Additionally, the remote and inaccessible locations of the study sites led to various kinds of infrastructure gaps that compromized the integrity of the samples, such as inconsistent access to electricity, or difficulties maintaining and repairing equipment. One written report recounted how after a power transformer explosion, a field staff had had significant difficulty sourcing funds for the repair and gaining access to a technician.

### Cultural barriers to communication

Field research staff included both native and non-native Khmer speakers. Nevertheless, the studies took place among minorities and remote populations, who often spoke a different language or dialect. This led to frequent reports of communication difficulties. Even when there were no language barriers, high illiteracy rates and poor malaria knowledge among these remote and under serviced populations resulted in communication challenges:

*“They say that malaria is not caused by the bite of the female mosquito of Anopheles; they say mostly it is because of the effect of environment changes, and they have incorrect knowledge about the supernatural spirits in the forests. This is my difficulty*.” *FGD25*

*“The causes of malaria for some patients are still not clear. Some communities mentioned that malaria is caused by cursed land, water and spirit: We have to explain to people not to be superstitious. They must not believe that malaria is caused by land or forest curse. They must have confidence in doctors.” IDI27*

Potential participants, therefore, often refused to join studies for a variety of reasons. Some did not believe the field staffs’ explanation for why they were sick. Others believed that anti-malarials would harm them or refused to provide blood samples. One field staff noted, *“We tell them they need medical treatment, but whether they agree or not is their decision, and we see that they decide to pray and believe in superstition.” FGD03*

Another explained, in regard to the collection of blood samples, “*They believe that the blood will not come back. It will be lost forever, and that will cause them tiredness or weakness.”* IDI11

Yet another said, *“when we start the survey, we provide a drug to the patient. People in the village feel hesitant to take it. They think it may cause cancer and in the future, after two years, they will die. So, we tried to have a meeting with them to explain that this drug is from the government. The government will not try to kill you.”* IDI10

Sometimes study staff faced opposition from local figures with competing interests, like traditional medicine practitioners or drug store owners: “*The drug seller at the private pharmacy sells an anti*-*malaria drug. The seller was saying that we were doing an experiment on the villagers, so he wouldn’t lose income.”* IDI11

These barriers made it challenging to engage in more complex discussions about malaria, and to explore the potential benefits of participating in research studies. They also complicated simple, routine processes like obtaining consent and administering questionnaires. Consequently, activities took more staff time than had been allotted in the planning stages, and resulted in delays and missed deadlines.

Some research staff noted that strong partnerships with local bodies had the potential to minimize mistrust and connect with potential participants more effectively. One proposed that “*to earn trust, we should have some people from local organizations accompanying us when we speak to new patients*.” IDI 05.

Others highlighted the need to spend time in communities to build rapport: *“No one will trust you if they only see you once or twice. Sometimes I spend my time with them in the evening. In the evening in the village they take traditional wine. I cannot refuse them; I just take a little bit to make them happy. It’s a kind of communication.”* IDI15

### Physical, emotional and financial strain to field workers

This study found that the conditions described above exacted a physical, emotional and financial toll on field staff. The conditions for many field workers were in themselves exacting. A study staff reported, *“we do the research in a different area. That’s why we need to live away from the family. My children always ask me when I am coming back, how long will it take.”*

Staff spoke of motor vehicle accidents in mountainous roads with heavy flooding. The pressure to reach sample sizes sometimes led to dangerous decisions: *“the survey had already finished, but we were missing participants. Then we decided to travel to another village, far. One foreigner and I went there. It was night and we needed to cross through the jungle and some streams to get to that village, so we could find some more participants. Later I learned that the whole way was full of landmines. My friend showed me the maps. There were so many landmines. I was very sorry that I led the foreigner there.”* IDI15

Many field staff felt overworked. One said, “*“I never take time off during the weekends. We have to do the research. When we have national holidays, the hospital staff take time off work. But actually, we don’t, except for Khmer New Year.”* Another was unable to take time off after a motorbike accident: “*It got slippery. I was driving a motorcycle to the field and I had an accident. I had to push the motorbike myself with so much pain in my shoulder and my leg for three kilometers, and get to the village to make contact with the village leader. The next day after the accident I still had to go back, in severe pain. So much pain. After three days, the pain was still going on. I could not walk properly but still I had to walk from house to house with pain in my leg and shoulder, to find recruits. This is the way we tried to recruit the patients.”* IDI23

High levels of stress also arose from difficulties meeting deadlines due to logistical and communication problems.

A further pressure was the need to balance field work with other work and family responsibilities. A large proportion of field staff, such as VMWs, were volunteers who did not receive financial compensation for their research-related tasks. Study staff reported having to choose between their other responsibilities and ensuring patient follow-up and continuity of care: “*They are so busy with their work. So am I. But I am strongly committed to being an MMW*”. IDI 06

The difficulty in maintaining this balance in a low-resource setting can be seen in the following exchange:

“*I. What is your challenge related to treatment?*

*P: It is difficult to follow up because they live far away. But I still ride my bicycle to see them until they recover.*

*I. Did you go to see them every day?*

*P. I follow up on the first day and last day and, sometimes, visit them 2 or 3 times again. My family doesn’t want me to go because there are no means to go.”* IDI23

At the same time, a significant proportion of research staff were hospital workers. For these field workers, neglecting their hospital responsibilities created a further ethical dilemma, as the hospital was understaffed, and research activities often required them to leave their post unmanned, potentially putting hospital patients at risk.

High rates of loss-to-follow-up were another source of emotional strain, as one field worker explained, “*for example, today we give him medicines, and the following day he disappears. And we are afraid that he takes medicines inappropriately*.” FGD15

Exclusion criteria also posed ethical dilemmas. A study staff noted, *“I have to exclude the pregnant ladies. And the tiny kids under one year. Also, the patients with severe disease. If that patient looks very pale, we exclude. And the very old, from 65 and above. Cambodian men 70 and above still go to the forest. If we exclude them, they will spread malaria.”* Similarly, patients with recrudescence were to be excluded and referred to their nearest health center. Research staff reported often breaking protocol and treating excluded patients, even those on their second and third recrudescence episode, at the study site on their own time; they knew that excluded patients were unlikely to be able to access adequate care otherwise.

Going above and beyond the scope of official duties was a common practice. One field worker said they had travelled for an entire day just to ensure that one patient took their medications and remained in the study. Another reported that they personally purchased a mobile phone for a study participant to support follow-up. A third routinely purchased rice for the poor in the villages to earn their trust. A field worker said: *“In the evening I spend some time with them, playing volleyball or snooker. We put down money for snooker but our team never wins. But we don’t care, we are building trust. This is from my own pocket.”* IDI12

One team of field workers resorted to publicly ingesting anti-malarials: “*When we provide the drug, we take the drug in front of them ourselves. Then they trust us. We split the team. If you are responsible for that village, you need to take the drug in front of them for three days. I did three full treatments in three months.”* IDI11

At the same time, staff expressed frustration because participants in whom they had invested so much time and even money would frequently be lost to follow-up or disqualified due to recrudescence (Table 1).

**Table 1:** Characteristics of studies included in content analysis. Adapted from [41]

Province where the research project was implemented	Institution carrying out the research project	Study methodology	Research topic	Data collection period	Frequency of reporting	Based
Pailin/Battambang	National Malaria Control Programme (CNM) International research institution	Cross-sectional study	Epidemiology and transmission	July 2012–Dec 2013	Quarterly	Field based
Pailin/Battambang	National Malaria Control Programme (CNM) International research institution	Cross-sectional study	Epidemiology and transmission	July 2012–September 2013	Quarterly	Field based
Pailin	National Malaria Control Programme (NMCP) Local research institution	Cross-sectional study	Health system (including private sector)	Jan 2013–Sep 2013	Quarterly	Cross-border based
Pailin/Battambang	National Malaria Control Programme (CNM) International research institution	Cross-sectional study	Surveillance	Jul 2013–Jan 2014	Quarterly	Health provider based
Pailin/Battambang/Pursat	National Malaria Control Programme (CNM) International research institution Local research institution	Clinical trial	Treatment	July 2014–March 2015	Monthly	Hospital based
Pailin/Battambang	National Malaria Control Programme (NMCP) Local research institution	Cross-sectional study	Surveillance	July 2012–July 2013	Quarterly	Field base and health centre based

### Limited avenues to report challenges

All written reports analysed were followed standardized formats, which research staff were required to fill out (Table 2). The quarterly reports contained a section called “Risk Reporting,” in which staff were to list any challenges they anticipated in the near future, assign a score meant to represent the likelihood of that challenge affecting the study, and detail what steps had been taken to mitigate the risk. If no challenges were predicted, the author would write “no risk reporting for this period”. Usage of this section was found to have the potential to mitigate damage. For example, reporting a potential flood which could cause delays led to the formation of an alternative plan that was implementation once flooding had subsided. Similarly, foreseen delays due to a national election campaign were mitigated by working closely with officials.

**Table 2:** Main themes of actual and predicted challenges listed in monthly/quarterly reporting

Recruitment and retention issues	Patients not from town so anticipated to be difficult to follow up People go back home for holidays and therefore difficult to recruit and follow up Low load of cases due to end of malaria season Private sector: patient treated in private sector before referral to study, lack of incentives for private providers to Many patients screened are migrants and cannot be included in studies Low numbers of border crossings High number of refusals Weekly target set to mitigate inability to reach sample size Gather more information on those who refuse to mitigate sampling bias due to refusals Lack of support from local authorities. Local authorities do not see malaria as a health problem anymore. There are other more pressing diseases Lack of cooperation from village chiefs
Environmental/external	Flooding National election campaign Unpredictability of border activities makes sampling difficult
Limited resources	Not enough staff to cover provincial level study Transport issues Expensive repair fees for hospital equipment compounds challenges created by power transformer explosion (Lab loses electricity) Sysmex machine out of service and needing engineer attention Unable to find reagents and samples not processed in time
Data quality issues	Difficulties in communicating with participants Delays in obtaining laboratory results Poor quality samples due to transportation issues e.g. freezing of samples, sample storage and analysis delayed due to blackouts in remote and rural areas, samples coagulated due to long travel distances and flooding during the rainy season
Staff/training	Delayed recruitment of assistance Training slow due to lack of staff Staff start losing their malaria diagnosis and treatment skills due to low malaria burden

Despite this attempt to predict future hypothetical challenges, the reports did not have a space where field staff could discuss actual challenges they had already faced in the study. Despite the lack of opportunity, some study staff added a “Challenges” section of their own accord (3 reports), and others inserted a “Challenges” subheading in the “Key Achievements” section (4 reports). Challenges were included more frequently and in a more detailed manner in monthly reports than in quarterly reports, and in general, monthly reports were longer and more complete than quarterly reports.

The written reports were the only official channel identified by this study through which challenges could be reported and documented.

## Discussion

The findings of this study shed a much-needed and often-neglected light on the issues faced by field staff conducting research in a malaria elimination setting. More importantly, however, they showed that these largely-overlooked perspectives can play a key role in maximizing the effectiveness of research studies, particularly critical studies required to reach the malaria endgame.

### Formal monthly reports are essential

The study found that written reports could be a very efficient tool for documenting field staff experiences, concerns and recommendations in an organized and standardized manner that could influence the design and conduct of future research studies. When instituting formal reporting, it would be crucial to include a “Challenges Faced” section; none of the reports analysed in the study contained such a section, but it was evident that field staff were eager to document their challenges. Additionally, a “Risk Reporting” section should be included in all reports, instead of only in some; when this section was used, barriers to achieving research goals were identified, and sometimes overcome. Finally, the experience of field study staff should be taken advantage of by providing a space for them to propose their own suggestions and solutions to problems. A system of formal reporting with these features would provide more comprehensive insight into logistical, communication and other difficulties that this study was only able to touch upon briefly. Gathering information on refusals to enroll in studies could also help to mitigate sampling bias within a study, and allow for measures to be put in place to reduce recruitment bias in future studies.

It was interesting that the monthly written reports analyzed in this study were more detailed than quarterly reports and contained more challenges and risk predictions, despite covering a shorter period of time. This could in part be due to personal preferences of the workers who filled out the reports. However, the brevity of quarterly reports could also be due to events having been forgotten or resolved by the time the report was written, as it would have been further removed from the relevant time period. It is, therefore, likely that monthly reporting would provide a more accurate understanding of field challenges than quarterly reporting.

While the reports analyzed in this study provided good insight, they had all been filled out by the study coordinator or study site principal investigator. Although the reports were meant to represent the study field staff as an entity, a measure of bias is unavoidable with a single author. It is therefore recommended that, in order to obtain a more comprehensive panorama of field challenges, several field workers in each study engage in regular reporting.

### Lessons learned from field workers should influence study protocols

It is currently uncommon for documented field challenges to be prioritized or taken into account in research protocols, even by experienced field researchers. Nevertheless, this study found that, had they been foreseen, a large portion of the challenges encountered by field staff could have been accounted for and avoided during the planning stages of the study. For example, accounting for delays caused by communication barriers when setting deadlines, or increasing the transportation budget to reduce the burden of long distances on field staff, could go a long way towards ensuring that study goals are met, and in turn significantly reduce physical, emotional and financial stress on field workers. The challenges captured in formal reporting should therefore be compiled into a dataset and used to inform the design of future research studies to prevent the same mistakes from being repeatedly. Operational research can yield innovative solutions to complex problems, such as loss-to-follow-up or inaccessible study sites that emerge in regular reporting but have no obvious immediate solution.

### Barriers to participant recruitment should be addressed

Participant recruitment was a recurrent topic in this study; it was hindered by a variety of factors, including logistical barriers, communication gaps, the socioeconomic context of patients, and the exclusion criteria of studies. It was also one of the primary sources of financial, emotional and physical strain to field staff.

The inability to reach the sample size required is an ever-present issue in malaria studies conducted in low endemic settings [30]. In Western Cambodia, where this study was conducted, the bulk of malaria incidence is found among MMPs, as is the case in most of the Greater Mekong Sub-region. Western Cambodia’s high volume of human population movement [22, 31–33] has been documented to contribute significantly to the spread of multidrug resistance. Despite all this, exclusion of MMPs is a commonly-accepted criterion for therapeutic efficacy studies, due to obvious difficulties with follow-up [14]. Including this key demographic in future drug studies will help to significantly reduce some of the difficulties around participant recruitment that field workers currently face. It will also be vital if Cambodia is to reach its goal of eliminating *P. falciparum* from the country by 2025 [33]; malaria elimination cannot occur as long as the most essential demographic is still being excluded from research studies.

Including MMPs in research studies will require innovative approaches to allow for follow-up given their mobility. The development of such approaches is sorely needed, as is noted in the WHO’s artemisinin resistance containment (ARCE) project. Operational research and lessons learned from similar epidemiological contexts, as well as lessons learned from local field staff, can help to inform this process. Currently, projects that aim to locate and map MMPs are being implemented in Cambodia and Vietnam [34]. Another possible solution was piloted in 2014 in Western Cambodia; MMPs who were recruited to participate in a study were provided with cellphones, which allowed the study team to stay in contact with participants after they had left the area. The study team also relied on Health Centers in other provinces to which study participants had migrated to collect samples and send them back to the study site. This method proved to be highly effective in providing follow-up to MMPs, but was discontinued due to the extensive resources it consumed.

Obstacles to participant recruitment could potentially also be reduced by addressing communication barriers. In this study, field staff mentioned that engaging with communities sometimes facilitated better communication with and easier recruitment of participants. There has been little research on how to most effectively engage communities in research [35]. Studies have shown that engaging and treating communities with respect results in more effective recruitment, more informed consent, and better relationships between research staff and study participants [35]. One suggested mechanism is a community advisory board (CAB), which is generally set up as a short-term initiative to inform a particular research project [35]. CABs are a popular mechanism to facilitate community engagement in externally-funded health research in low and middle-income countries, serving as a liaison between research teams and research participants [36, 37]. They can also play a complementary role to ethics and scientific committees, and have the potential to improve the ethics of a project, including informed consent [35]. Additionally, local community leaders in Western Cambodia understand their communities and are often well respected. They can play a key role in providing education to potential participants, and otherwise mitigating communication barriers [38]. This would in turn reduce delays, and the stress they cause. In this societal context, the impact of fostering strong relationships with local leaders should not be underestimated.

### Overlaps between clinical care and research warrant careful consideration

Malaria research in resource-poor settings often involves an overlap between research and clinical care [39]. Field research teams are often composed of health workers providing care to the community, such as the Pailin Referral Hospital employees and the VMWs who participated in this study. On the one hand, there are several advantages to this practice; qualified research staff are hard to come by, and health workers are already immersed in their communities and have the best access to potential study participants. It seems like a natural fit. Nevertheless, health workers in this study had difficulty managing their jobs and research duties. Several recounted abandoning their posts, and therefore depriving an already resources-poor community of health service in order to ensure the success of the study. They also noted having emotional difficulties with this ethical dilemma. The overlap between research projects and healthcare services could also act as an inducement, raising the potential for ethical issues of voluntariness to arise [40]. Ethics committees may wish to take this into account when approving research studies that use health workers as field staff.

## Conclusions

This is the first study that documents the challenges faced by field staff in conducting studies in malaria elimination settings many which are unique to this context. In order to improve the efficiency and quality of future studies, learning from and taking into account the experiences of study field staff is crucial. Lessons learned from this study can help to address some inefficiencies currently plaguing research studies in drug-resistant malaria elimination settings, and, most importantly, to capture field study staff experience as effectively, so that a more extensive dataset can be used to inform future study designs.

## Additional file

**Additional file 1: Table S1.** Provinces, ODs and HCs visited by the quantitative field team in zones 1 and 2 of the containment project. **Table S2.** Sociodemographic characteristics of 197 VMW and MMWs.

## Abbreviations

CAB: community advisory board
FGDs: focus group discussions
IDIs: in-depth interviews
MMPs: mobile and migrant populations
MMWs: mobile malaria workers
VMWs: village malaria workers
