# Supplementary material for: Maximizing research study effectiveness in malaria elimination settings: a mixed methods study to capture the experiences of field-based staff
Source: Malar J. 2017 Sep 11;16:362. doi: 10.1186/s12936-017-2016-4 (PMC5594431; doi:10.1186/s12936-017-2016-4)
Supplement: Supplementary file 1 — Additional file 1: Table S1. Provinces, ODs and HCs visited by the quantitative field team in zones 1 and 2 of the containment project. Table S2. Sociodemographic characteristics of 197 VMW and MMWs. [file 12936_2017_2016_MOESM1_ESM.docx]

Table S1: Provinces, ODs and HCs visited by the quantitative field team in zones 1 and 2 of the containment project.

| Zone | Province | OD | HC |
| --- | --- | --- | --- |
|  |  |  |  |
| Zone-1 | Kampot | Chhouk | 1-Traping Raing |
|  |  |  | 2-Chres |
|  |  |  | 3-Sre Chheng |
|  |  |  | 4-Chumpou Voan |
|  | Pursat | SampovMeas | Pramuoy |
| Zone-2 | Battambang | MoungRussey | 1-Preay Tralach |
|  |  |  | 2-Koh Karlor |
|  |  |  | 3-Prek Chick |
|  | Kampong Speu | Kampong Speu | 1-Treang Trayeong |
|  |  |  | 2-Talat |
|  |  |  | 3PreychumpoumeanAng |
|  | Preahvihear | Thbengmeanchey | 1-Thbeng meanchey |
|  |  |  | 2-Phnum Dek |

Table S2: Sociodemographic Characteristics of 197 VMW and MMWs

| Sociodemographic Characteristics | TOTAL | |
| --- | --- | --- |
|  | N | % |
| Gender  Male  Female | 115  82 | 58  42 |
| Age in years (mean, SD) | 35.5, 11.62 | |
| Primary profession  Farmer  Labourer  Fisherman  Merchant/Seller  Housewife  Other * | 182  2  1  3  3  6 | 92.4  1.0  0.5  1.5  1.5  3.1 |
| Highest level of education  Never attended school  Some primary  Completed primary (grade 6)  Some secondary  Completed secondary (grade 12)  More than secondary | 71  22  97  7  -  - | 36.0  11.2  49.2  3.6  0  0 |
| Marital Status  Single—never married  Married/living with someone as married  Widowed | 27  163  7 | 13.7  82.7  3.6 |
| Ethnic group  Khmer  Cham  Kuoy  Other ¥ | 193  1  2  1 | 98.0  0.5  1.0  0.51 |
| Respondents role  VMW  MMW | 163  34 | 82.7  17.3 |
| Length of time working as a VMW/MMW  < 1 year ago  1 year ago  2 years ago | 7  26  164 | 3.5  13.2  83.3 |

* Others included: environmental officer, primary school teacher, security guard, teacher, veterinarian
¥ Other included: minority group (unspecified)
